# The atypical quorum sensing system of classical *Brucella* species

**DOI:** 10.1128/jb.00388-25

**Published:** 2025-11-12

**Authors:** Mitchell T. Caudill, Clayton C. Caswell

**Affiliations:** 1 Center for One Health Research, Department of Biomedical Sciences and Pathobiology, VA-MD College of Veterinary Medicine, Virginia Tech 229659, Blacksburg, Virginia, USA; Geisel School of Medicine at Dartmouth, Hanover, New Hampshire, USA

**Keywords:** *Brucella*, quorum sensing

## Abstract

*Brucella* species are notorious pathogens of animals and humans and cause significant morbidity and economic losses globally. These hardy bacteria have evolved to survive and replicate in host cells, particularly macrophages, and have developed a specialized quorum sensing system that is essential for navigating intracellular life. Moreover, successful infection of the host is dependent upon elements of the *Brucella* quorum sensing system. While quorum sensing is a thoroughly well-defined process in many Gram-negative bacteria, several unique features in the quorum sensing pathway have evolved that set *Brucella* apart from more established model organisms. The current review is aimed at describing the paradigmatic aspects of *Brucella* quorum sensing, while also underscoring the nuance and distinctiveness of quorum sensing in the brucellae, and we discuss important questions that remain unanswered in the field.

## INTRODUCTION

The genus *Brucella*, both historically and presently, has a contentious taxonomy. Because the genus contains species of global public health concern, clarity for public health messaging largely prevailed over desires for a unified, nonparaphyletic phylogeny (1, 2). *Brucella melitensis*, *B. suis*, and *B. abortus* are zoonotic and highly pathogenic causes of the disease brucellosis, while *B. canis*, *B. ceti*, and *B. pinnipedialis* strains are presumptively pathogenic in humans but less frequently observed epidemiologically. These species, along with the sheep pathogen *B. ovis*, form the informal clade of “classical” *Brucella* and are closely related (3–6). Other *Brucella* species, including those of the former *Ochrobacterum* genus, show higher genomic and phenotypic diversity and are informally termed the “non-classical” or “atypical” species (7–10).

**Fig 1:**
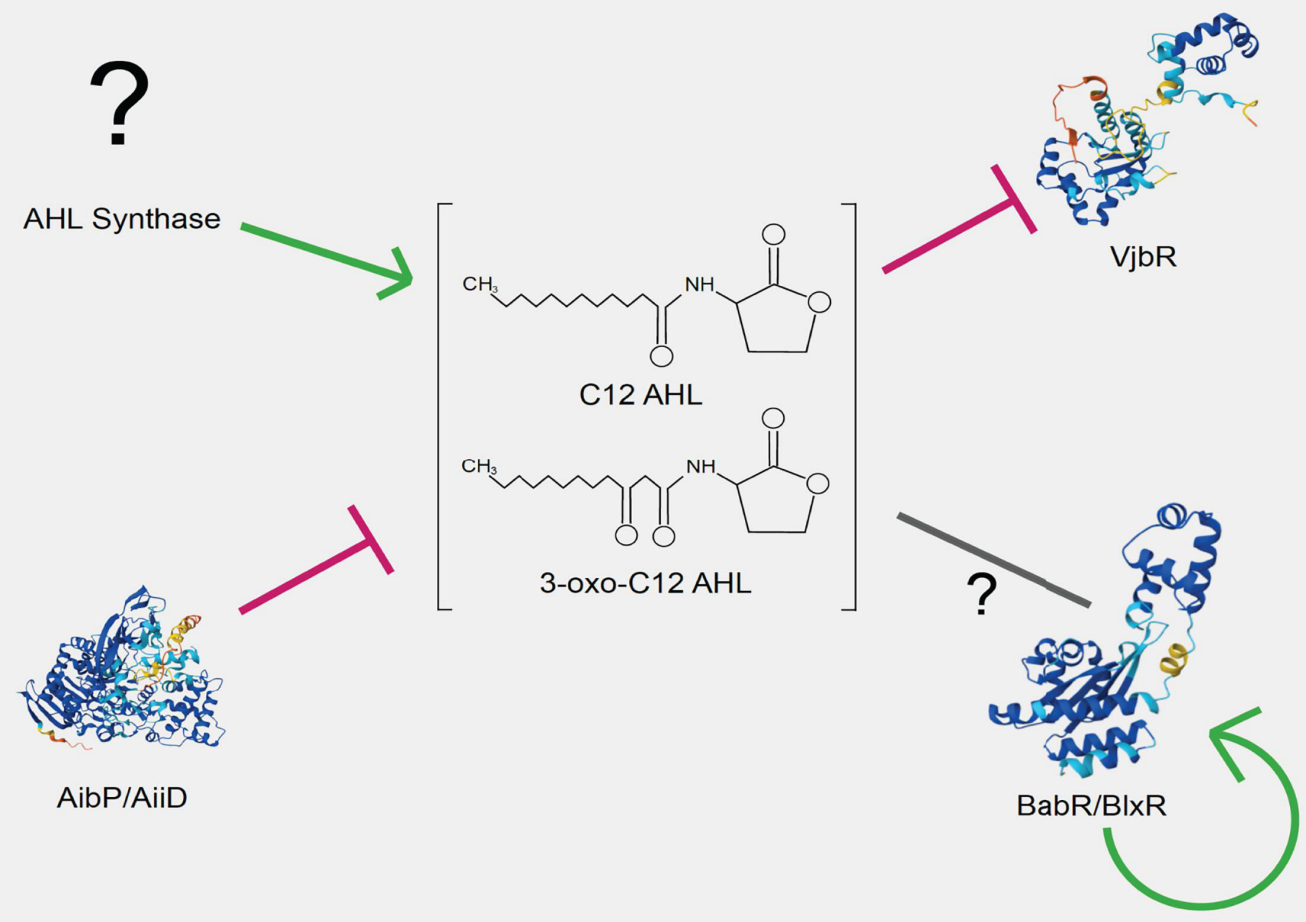
The components of *Brucella’s* quorum sensing system (QSS). The molecular components of Brucella’s quorum sensing system are presented. The unknown AHL synthase synthesizes C12 AHL and 3-oxo-C12 AHL, while the acylase protein AibP/AiiD degrades these signals. AHL, in turn, inactivates the function of VjbR by dissociating it from DNA. AHL’s exact effect on BabR/BlxR is not definitively known, but BabR does autoregulate its own transcription. Displayed proteins are predictions of structure generated using AlphaFold within UniProt (11). Regarding the protein structures, the colors correspond to the confidence of the predicted structures as calculated by pLDDT: dark blue = very high (pLDDT >90); light blue = confident (90 > pLDDT > 70); yellow = low (70 > pLDDT > 50); orange = very low (pLDDT <50).

This review focuses on the quorum sensing system (QSS) of the classical species, which are species of major public health concern. The QSS has been of special interest to the field of *Brucella* researchers as a means of developing next-generation live-attenuated vaccines, as well as for better understanding the regulation of *Brucella*’s pathogenesis and virulence programs. The QSS circuitry in *Brucella* consists of an unknown acyl-homoserine lactone (AHL) synthase, which produces dodecanoyl homoserine lactone (C12) and 3-oxo-dodecanoyl homoserine lactone (3-oxo-C12), an acylase, AibP/AiiD, which breaks down the AHL signal, as well as two LuxR-type regulators, VjbR and BabR/BlxR (Fig. 1; Table 1). Each of these components is discussed in more detail below.

**TABLE 1:** Genetic components of the QSS

Species	Locus tag for:		
	*aiiD*	*vjbR*	*babR*
*B. abortus* BAB	BAB2_1048	BAB2_0118	BAB1_0190
*B. abortus* BAB_RS	BAB_RS31290	BAB_RS26915	BAB_RS16810
*B. melitensis* BME	BMEII0211/BMEII0212	BMEII1116	BMEI1758
*B. melitensis* BME_RS	BME_RS11225/BME_RS11230	BME_RS15625	BME_RS08725
*B. suis*	BR_RS15145	BR_RS10615	BR_RS00880
*B. ovis*		BOV_RS10895	BOV_RS00900
*B. canis*	BCAN_RS15170	BCAN_RS10670	BCAN_RS00895
*B. ceti*	ACL594_RS12975	ACL594_RS14135	ACL594_RS06055
*B. pinnipedialis*	BPI_RS15770	BPI_RS10950	BPI_RS00870

Given the high genomic similarity between the classical species, the genetic basis for the differences in observed epidemiological phenotypes (e.g., the degree of virulence and the typical mammalian host of infection) has been of longstanding interest and without clear basis (12–15). Regulatory action by the QSS may drive some of these phenotypic differences, and as we review the QSS, we will emphasize where phenotypic differences have been observed between the classical species.

The gene annotations for the components of the QSS for each of the classical *Brucella* species are provided as a reference, and each annotation is noted by the locus tag for each given *Brucella* species. The specific NCBI RefSeq assemblies for each reference genome are as follows: *B. abortus* 2308 (GCF_000054005.1); *B. melitensis* 16M (GCF_000007125.1); *B. suis* 1330 (GCF_000007505.1); *B. ovis* ATCC 25840 (GCF_000016845.1); *B. canis* ATCC 23365 (GCF_000018525.1); *B. ceti* DDE002 (GCF_047324105.1); *B. pinnipedialis* B2/94 (GCF_000221005.1).

## COMPONENTS OF THE QSS

### AHL synthesis and breakdown

The mechanism of AHL synthesis remains unknown in *Brucella* strains. *Brucella* genomes lack homologs to either LuxI- or LuxM-type synthases, which are classical synthases for acyl-homoserine lactones (16, 17). HdtS has been suggested as a potential third class of AHL synthase (18), and *Brucella* strains do encode a protein with partial homology (i.e., BAB1_1994 in *B. abortus* 2308); however, there are no experimental indications that BAB1_1994 or its homologs in other species possesses AHL synthase activity. Moreover, our group and others have attempted to identify the mechanism of AHL synthesis in *Brucella*, and unfortunately, thus far have failed (19, 20). For the authors, and likely for many others as well, the identity, regulation, and mechanism of action of the *Brucella* AHL synthesis pathway(s) remain some of the most intriguing open questions in the field.

An AHL signal generated by *Brucella* was originally detected by Taminiau et al. using mass spectroscopy on extractions from *Brucella melitensis* spent culture (21). Generating sufficient material to identify the C12 signal required extraction from 18L of culture, a massive amount given the infectious dose of *B. melitensis* (i.e., approximately 10 CFU). These analyses also indicated smaller amounts of 3-oxo-C12 in the supernatant and that the amount of C12 produced varied with the growth medium used, but they did not report a specific mechanism altering the production of C12.

In a follow-up to these findings, Terwagne et al. estimated the intrabacterial concentrations of C12 at most 1 nM and 3-oxo-C12 at 100 pM during *Brucella melitensis* growth in rich medium using an exogenous reporter system that linked the *Pseudomonas* LuxR, LasR, to the expression of GFP (22). These estimated concentrations are quite low when compared to the effluent values (as opposed to biofilm values) for the prototypical QS pathogen *Pseudomonas aeruginosa* where AHL concentrations are in micromolar quantities (23). Nonetheless, it is worth noting that some other members of the *Hyphomicrobiales* also produce relatively low levels of homoserine lactones. Examples include stem-nodulating strains of *Bradyrhizobium* that generate nanomolar concentrations of AHL (i.e., 30–65 nM) (24), and *Bradyrhizobium japonicum* USDA110 was shown to produce approximately 5 nM of AHL in liquid culture (25). Even so, these examples of low-level AHL production are significantly higher than the estimated concentrations of AHL produced by *B. melitensis* during culture in rich medium, and it remains to be determined if *Brucella* AHL production is substantially elevated in cells grown in other media or lifestyle contexts (e.g., within the host cell vacuole).

Given the lack of AHL synthase homologs, some have suggested that *Brucella* lacks AHL synthesis entirely and that the LuxR are orphaned (26, 27). We do not find this likely, given the phenotypic changes when the AHL-binding domain of VjbR is mutated or the AHL-degrading acylase is removed in *B. melitensis* (discussed further below). As such, we find it likely that endogenous AHL is generated and sensed, both during culture and infection. The LuxR proteins also respond to the AHL signal at the levels estimated (28), and it is unclear how the bacterium would sense AHL in its multimembrane-enclosed vacuolar environment, except through endogenous production. The direct evidence for AHL production in *Brucella* species other than *B. melitensis* is lacking; however, our laboratory has found that wild-type *B. abortus* can activate an exogenous AHL reporting system (data unpublished). As such, the relative levels of production of the AHL signals between the species are unknown and bear further investigation.

In addition to synthesis, *Brucella* produces an AHL acylase variously called AHL inactivating *Brucella* protein (AibP) and AiiD (unknown meaning) that breaks down the AHL signal (20, 28). As shown by Terwange et al., the levels of C12 peak in the mid-exponential phase and then decline throughout the stationary phase in response to AiiD activity (22). This capping of the maximum C12 signal and decline is due to the action of the acylase, and deletion of the acylase results in steady accumulation of C12 in line with cell density. The signals and mechanisms controlling *aiiD* expression are not known; however, transcript expression level peaks in the late exponential phase. Despite the role of C12 and the QSS in regulating virulence gene expression, deletion of *aiiD* does not result in attenuation of bacterial infection for *B. melitensis* in either macrophages or a mouse model infection. Homologs of AiiD are present in all the classical species, except *B. ovis*.

### LuxR regulators

Two LuxR-type regulators are present in *Brucella* species: vacuolar hi-jacking *Brucella* regulator (VjbR) and *Brucella abortus* regulator (BabR) also known as BlxR (29). Several other LuxR-type regulators are annotated within the *Brucella* pangenome, but these proteins lack AHL-binding domains and presumably use different ligands to regulate their DNA-binding via the LuxR winged helix-turn-helix domain. Both VjbR and BabR act transcriptionally in response to C12 and 3-oxo-C12 and have broad genetic regulons (19, 30–35).

VjbR is by far the most well-studied component of the *Brucella* QSS, likely due to its importance in stimulating the expression of critical virulence factors including the Type IVb secretion system (T4SS) and its secreted effectors, the flagellum, and outer membrane proteins. VjbR was initially identified in a transposon screen for insertion mutants defective in replication inside macrophages. It was named for its criticality in ensuring rerouting of the *Brucella*-containing vacuole out of the autophagy pathway to allow bacterial replication during intracellular infection (35). All evidence indicates that VjbR is a dissociative LuxR, with the presence of AHL signal abrogating its native DNA-binding capability, and this relationship has been shown for the *virB* promoter of the T4SS (19, 30, 36).

The spatiotemporal regulation of *vjbR* expression during infection has not been well characterized. Across one or more *Brucella* species, transcript levels of *vjbR* are indirectly regulated by several transcription factors including the following: transcriptional regulators BabR, OtpR, and HutC (30, 34, 37–39), sigma factor RpoE1 (40), the Lon protease (41), and the sRNA BmsR1 (42). Direct mechanistic regulation (i.e., direct binding to a promoter or *vjbR* transcript) has been shown for the HNS-like protein MucR and the transcriptional regulators BvrR and ArsR2 (43–47). In addition, it has been shown that translation of the transcript of *vjbR* is linked to low pH and urocanic acid (48). Turnover rates for VjbR protein are unknown. The specific links of VjbR to virulence are discussed further below, but linking the duration and activity of VjbR to specific events during infection will be a major step in understanding how *Brucella* species regulate their virulence program to maintain survival in the host.

The quest for a vaccine capable of providing safe, protective immunity against *Brucella* infection has been a long-term goal of agricultural producers and human public health (49–51). The major successful vaccines have been live-attenuated strains for livestock developed through passage. While these vaccines have efficacy in reducing livestock abortion, the safety profiles are poor, with all the live-attenuated vaccines capable of causing infections in humans, if exposure occurs. Given the high attenuation of Δ*vjbR* strains, an effort has been made to test these strains as purposefully designed live-attenuated vaccines (32, 52–55). Δ*vjbR* strains have been made in nearly all the classical *Brucella* species with promising results for protective immunity. While a full review of *Brucella* vaccinology is outside the scope of this review, it appears that vaccines based on VjbR show promise as a next-generation *Brucella* vaccine.

The other LuxR regulator, BabR, is much less characterized than VjbR. There is tentative evidence that BabR is also a dissociative LuxR given that in the screen that led to its discovery, it was binding DNA without AHL present, though this occurred via heterologous expression in *E. coli* (20). Regulation of *babR* expression is also poorly explored. Indirectly, Lon protease, the sRNA chaperone Hfq, and transcriptional regulators VjbR, OtpR, and HutC all play a role in *babR* expression (30, 34, 38, 39, 56). Direct regulation of *babR* transcription has been shown for BabR itself in an autoregulation role, as well as for the HNS-like MucR (30, 33, 43, 57). Significantly more work is needed to determine the pattern and duration of BabR activity.

## QSS AND VIRULENCE

### AHL

Specific links between levels of AHL and *Brucella* virulence have been difficult to test. Presumably, any effects of AHL on virulence are mediated by the LuxR proteins, though a recent analysis by our laboratory found potential for LuxR-independent transcriptional changes in response to AHL in cells cultured in rich medium (30). Additionally, there remains open potential for *Brucella*-generated AHL to directly interact with host membranes, which has been observed for some bacteria but is not documented in *Brucella* (58, 59).

Since the AHL synthase is not known, it is not possible to genetically delete the mechanism, but other approaches have been used to examine the effects of loss of AHL on bacterial physiology. Godefroid et al. overexpressed the AHL-acylase AiiD, while Uzureau et al. abrogated VjbR’s capability to bind AHL, locking it into a function “on” state (28, 60). Both experiments were performed in *B. melitensis* and resulted in clumping of cells in culture and bacterial production of concanavalin A (ConA) lectin-positive staining α-mannopyranosyl and/or a-glucopyranosyl exopolysaccharide. Further analysis of the AiiD-generated exopolysaccharide revealed it to be primarily mannose with traces of galactose, glucosamine, and glucose. However, there are two caveats that need to be considered regarding the composition of the exopolysaccharide: (i) only the strain carrying the *aiiD* over-expression plasmid was analyzed, while the wild-type strain and the wild-type strain harboring the empty plasmid were not evaluated; and (ii) the medium used to culture the *aiiD* over-expressing strain contained extracts of yeast, which contain mannans in the cell wall. Therefore, it is possible that the exopolysaccharide identified was derived from yeast components, rather than being of bacterial origin. The abrogation of VjbR’s capability to bind AHL was achieved via mutagenesis of a critical amino acid within the AHL-binding region and curiously only resulted in the clumping phenotype for *B. melitensis,* but not *B. abortus*. Both papers arose from the laboratory of Jean-Jacques Letteson, which noted that the AiiD overexpression resulted in “larger and more stable clumps” (28).

Godefroid et al. also observed DNase I-responsive effects to the *B. melitensis* clumps, suggesting DNA forms a component of the matrix, and noted outer membrane vesicles in electron micrographs. Taken together, they cautiously stated the potential for *B. melitensis* to form “biofilm-like structures,” which would indicate that AHL may have a role in allowing the formation of such structures. Anecdotally, the claim that classical *Brucella* species are able to form biofilms remains controversial and contested. This is likely due to the above structures and similar clumping phenotypes occurring in artificial genetic backgrounds or in conditions that are likely not achieved physiologically. Additionally, *Brucella* strains can naturally dissociate into “rough” LPS variants that are receptive to crystal violet, requiring careful quality control for indirect crystal violet assays for biofilms that are often not noted within the *Brucella* biofilm literature. All of this is to say that the role of AHL in shaping *Brucella* virulence, with or without biofilms as a component, remains minimally explored.

### VjbR

VjbR is directly linked to the expression of the T4SS and the flagellum, *Brucella’s* two extracellular structures. Direct VjbR binding sites on the genome were documented in a ChIP-Seq approach by Kleinman et al., which found an asymmetric 16 bp consensus binding sequence motif (31). The links between VjbR, the VirB T4SS, and its effectors have been extensively documented, and VjbR binds directly to the *virB* promoter (31). Given that the *virB* operon is tightly regulated by other transcriptional regulators, Δ*vjbR* strains do not show a complete abrogation of T4SS expression, but there is a marked decrease in expression at the population level of T4SS and its effectors that contributes to the attenuation of Δ*vjbR* strains (36, 61).

VjbR also regulates flagellum expression through promotion of FtcR, which directly controls the expressions of genes encoding flagellar proteins (62). The role of the flagellum in *Brucella* infection remains obscure, but it has been reported that a *B. melitensis* strain lacking the ability to generate a flagellum is attenuated for infection (63). However, deletion of the three flagellar loci in *B. ovis* did not result in virulence deficiencies of the bacteria in a mouse model of infection (64). Additionally, comparative genomic analyses have revealed that many of the classical *Brucella* species lack certain flagellar genes and/or retain genomic elements of flagellar genes as pseudogenes (65), leading to the possibility that many strains do not possess the ability to produce a flagellum. Collectively, the precise role of the flagellar system across *Brucella* species is enigmatic, but there is a clear connection between VjbR and flagellar gene expression in some species, pointing to another potential role for the QSS in delineating the epidemiological phenotypes observed.

The broader, more indirect, roles that VjbR may play in metabolism and preparation of the bacterium for host infection are difficult to generalize due to the lack of standardized experiments across *Brucella* species, growth condition, or assay type. Transcriptomic or proteomic measurements of multiple species Δ*vjbR* strains in the same conditions, or even a single species across multiple conditions, would greatly aid in expanding the indirect roles of VjbR in shaping the metabolic and phenotypic states outside the direct expression of extracellular structures.

### BabR

Until a ChIP-Seq or similar experiment is performed to define the direct regulatory targets of BabR and test the effect of those targets with and without AHL, the exact mechanism of regulation of BabR on virulence factors will remain unknown. Nevertheless, BabR does perform functions during the infection cycle of *Brucella*. Deletion of *babR* results in attenuation of infection in an NRAMP +mouse model, and a recent study by our laboratory found it supports infection in the absence of VjbR and likely makes a minor contribution to virulence (30, 33). The ways in which BabR contributes to virulence are difficult to dissect due to the same lack of standard assays as VjbR. Targeted studies of BabR’s regulatory functions would greatly expand knowledge of the *Brucella* QSS and the interactions between the two LuxR proteins.

## CELL TRAFFICKING AND THE QSS

Given the close interconnection between the QSS, T4SS, intracellular trafficking, and the bacterial replication cycle, it is worth commenting on the known interactions and gaps in knowledge. Molecular details of intracellular trafficking have been thoroughly reviewed elsewhere (66, 67). The entry and exit mechanisms of *Brucella* remain incompletely defined, and the majority of studies have focused on naïve bacteria (i.e., those coming directly from laboratory culture) interacting with either macrophage or epithelial cell lines. *Brucella* strains egressed from intracellular passage are more infectious than naïve cells, though the mechanisms for this phenotype are not well defined (68).

For naïve cells, the QSS does not seem to play a role in cell entry, with QSS mutant strains showing no defect in the number of cells reaching the intracellular compartment (32, 33, 35). Instead, the attenuation in survival for Δ*vjbR* strains likely occurs during the transition of the endosomal *Brucella*-containing vacuole (eBCV) to the replicative BCV (rBCV), given that these dynamics occur with disrupted Type IV secretion (69). A careful study of the dynamics of vacuolar trafficking in Δ*vjbR* strains, particularly documenting whether those that do replicate express the T4SS in spite of the lack of VjbR, would be interesting and help confirm the assumption that much of the attenuation of Δ*vjbR* strains in the macrophage model occurs due to lack of T4SS expression.

Following replication, *Brucella* bacteria enter an advanced *Brucella*-containing vacuole (aBCV) and ultimately egress from the cell. Both mitophagy and multi-vesicular bodies have been implicated in *Brucella’s* egress, and the exact process of bacterial exit is unknown (70, 71). The QSS does appear to have a role in preparing egressing cells to reinfect and begin the cycle anew. After treating *Brucella* reaching the aBCV with C12 AHL, Altamirano-Silvia et al. found the bacteria were less adherent in a new infection cycle and fewer bacteria entered the cell and that this phenotype was likely independent of the Type IV secretion system, as tested by using a specific T4SS inhibitor (69). From this, we can surmise that the QSS, and VjbR in particular, is active in the aBCV and functionally prepares bacteria for reinfection.

Many gaps in knowledge exist regarding cell trafficking and the QSS. Aside from the limited cases discussed above, the spatiotemporal expression patterns of the QSS have not been well correlated to the various steps of the intracellular infection cycle. The relative levels of AHL throughout the cycle have only been indirectly detected via bacterially expressed reporter systems (22), and there have been no reports that seek to quantify the concentrations of free AHL in the various compartments and vacuole. Given that the AHL synthase is unknown, its expression and translation levels are also unknown. In terms of the intracellular trafficking of the brucellae, the activity levels of VjbR are poorly documented, and the role of BabR is unknown. Carefully documenting the QSS components during intracellular infection may greatly aid in explaining the systemic infection level phenotypes and attenuation, as well as aid in generating genetic interventions that increase immune protection of QSS mutant-based vaccines.

In addition to these fundamental questions regarding spatiotemporal expression, on a more fundamental level, it remains fundamentally unclear whether the QSS actually functions to sense quorum during the intracellular infection. Briefly, the phenomenon known as “quorum sensing”—meaning the phenomenon of cells responding to a self-generated chemical signal in a density-dependent manner—was originally termed autoinduction but was interpreted as a social phenomenon, implying the behaviors exhibited were collective in nature (72, 73). The term “quorum sensing” was provided upon documentation that the phenomenon was widely distributed in Gram-negative bacteria, with species each using the system to coordinate bacterial-host interactions (74). The term “quorum” implied that the bacteria were waiting until sufficient group numbers were generated in order to engage in a given group behavior, analogous to quorum in a meeting governed by parliamentary procedures.

This interpretation of data was later challenged with the alternate intellectual framework that proposed an individual bacterium may simply be sensing the diffusion rate of environment, with the corollary that it should be theoretically possible for a single bacterium to autoinduce its “quorum” system (75). This was subsequently borne out in experiments which isolated an individual Gram-positive bacterial cell (*Staphylococcus aureus*) and found autoinduction of QSS, and ultimately a rebuttal from the social interpretation that perhaps a “quorum of one” is possible (76, 77). As such, it is possible that, in some situations, the AHL Gram-negative QSS is also governing the actions of an individual bacterium sensing chemical diffusion of its surrounding, while also providing a means of controlling collective behaviors when bacteria are interacting in higher-density populations.

In the context of infection and natural lifestyle, it remains unknown for *Brucella* whether a bacterium produces AHL that is in turn sensed by other *Brucella* bacteria. The number of bacteria within the vacuole through the various stages is fairly small, and only 10–20 bacteria per cell are typically observed intracellularly (78, 79). The exception to this is the placenta, where individual trophoblasts can have over 100–200 bacteria per cell (80, 81). Whether each bacterium produces AHL sensed by itself, if the signals are density-dependent intracellularly, or whether only a percentage of the population produce the AHL signal for all members of the community is unknown. As such, it is unclear whether the bacterium is autoinducing or, indeed, quorum-sensing. Resolving these questions will require documenting the AHL synthesis mechanism or finding some other means of inhibiting C12 synthesis at the level of the individual bacterium.

## QUORUM SENSING IN OTHER CONTEXTS

In addition to typical systemic infection and intracellular trafficking, two other infection contexts are worth considering in regard to the QSS and are discussed below.

### Endocarditis

In systemic brucellosis, few cases can result in vegetative endocarditis. As endocarditis caused by other bacterial pathogens is often associated with biofilm formation and adhesion mechanisms that require efficient quorum sensing systems, it is possible that the QSS aids in mediating *Brucella*-associated endocarditis (82). The exact prevalence of endocarditis linked to *Brucella* infection is difficult to determine but is commonly reported as between 1% and 3% and has likely declined in contemporary settings compared to historical prevalence due to a concomitant decrease in cases of rheumatic fever damaging heart valves and predisposing to valvular presentations (83–85). Endocarditis, nevertheless, represents a clinically more challenging presentation and, from a bacteriological perspective, is a unique environment that raises the potential for biofilm or biofilm-like development and the accumulation of AHL signal. To our knowledge, the vegetative lesions of brucellosis have not been characterized chemically or biologically with modern techniques, and no endocarditis model for brucellosis has been developed. Whether the QSS promotes persistence in the valvular lesion and the relative levels of AHL and LuxR within the vegetation are intriguing questions.

### Implant-associated infections

In countries with endemic brucellosis, implant-associated infections have been observed in both joint- and soft-tissue devices or materials (see (86–88) as examples). Similar to endocarditis, implant infections are more clinically challenging presentations and offer a unique environment for the bacteria. *Brucella* species have known osteoarticular predilections and are well adapted to manage the immune environment of the bone and joint (89–91). It is likely that many of the same genetic programs used in bone and joint infections are used by *Brucella* in implant infections, but again, implants represent a potential for the use of biofilm or biofilm-like as a mechanism of immune evasion and persistence. Investigating these implants for the role of QSS may reveal new aspects of the system that may aid in clinical management of these challenging cases.
